# Prevalence and seasonality of common viral respiratory pathogens, including Cytomegalovirus in children, between 0–5 years of age in KwaZulu-Natal, an HIV endemic province in South Africa

**DOI:** 10.1186/s12887-018-1222-8

**Published:** 2018-07-21

**Authors:** Temitayo Famoroti, Wilbert Sibanda, Thumbi Ndung’u

**Affiliations:** 1 Department of Virology, National Health Laboratory Service, Nelson R Mandela School of Medicine, University of KwaZulu-Natal, Durban, KwaZulu-Natal South Africa; 2 Biostatistics Unit, School of Nursing and Public Health, College of Health Sciences, University of KwaZulu-Natal, Durban, KwaZulu-Natal South Africa; 3 HIV Pathogenesis Programme, Doris Duke Medical Research Institute, Nelson R Mandela School of Medicine, University of KwaZulu-Natal, Durban, KwaZulu-Natal South Africa

**Keywords:** Children, Respiratory virus, Seasonality, South Africa

## Abstract

**Background:**

Acute respiratory tract infections contribute significantly to morbidity and mortality among young children in resource-poor countries. However, studies on the viral aetiology of acute respiratory infections, seasonality and the relative contributions of comorbidities such as immune deficiency states to viral respiratory tract infections in children in these countries are limited.

**Methods:**

A retrospective analysis of laboratory test results of upper or lower respiratory specimens of children between 0 and 5 years of age collected between 1st January 2011 and 31st July 2015 from hospitals in KwaZulu-Natal, South Africa. Respiratory specimens were tested for viral respiratory pathogens using multiplex polymerase chain reaction (PCR), HIV testing was performed either by serological or PCR methods. Cytomegalovirus (CMV) respiratory infection was determined using the CMV R-gene PCR kit.

**Results:**

In total 2172 specimens were analysed, of which 1175 (54.1%) were from males. The median age was 3.0 months (interquartile range [IQR] 1–7). Samples from the lower respiratory tract accounted for 1949 (89.7%) of all specimens. Respiratory multiplex PCR results were positive in 834 (45.7%) specimens. Respiratory syncytial virus (RSV) was the most commonly detected virus in 316 (32.1%) patients, followed by adenovirus (ADV) in 215 (21.8%), human rhinovirus (Hrhino) in 152 (15.4%) and influenza A (FluA) in 50 (5.1%). A seasonal time series pattern was observed for ADV (winter peak), enterovirus (EV) (autumn), human bocavirus (HBoV) (summer), and parainfluenza viruses 1 and 3 (PIV1 and 3) (spring). Stationary or untrended seasonal variation was observed for FluA (winter peak) and RSV (summer). HIV results were available for 1475 (67.9%) specimens; of these 348 (23.6%) were positive. CMV results were available for 714 (32.9%) specimens, of which 416 (58.3%) were positive. There was a statistically significant association between the coinfection of HIV and CMV with ADV.

**Conclusions:**

In this study, we identified the most common respiratory viral pathogens detected among hospitalized children in KwaZulu-Natal. The coinfection between HIV and CMV was found to be associated with an increased risk of only adenovirus infection. Most viral pathogens showed a seasonal trend of occurrence. Our data has implications for the rational design of public health programmes.

## Background

Respiratory tract infections are common in children and account for significant cases of absenteeism from school, hospitalization and sometimes death [1]. Viruses are a leading cause of these infections in children under 5 years of age and are associated with significant morbidity and mortality [2, 3]. Among children aged 1–59 months acute respiratory infection, diarrhoea, and malaria are the leading cause of death with over 15% caused by acute respiratory tract infection (ARTI) [4]. It is estimated that up to 53% of infants will have a viral respiratory tract infection in the first year of life and about 3% of children less than 1 year of age may require hospitalization with moderate or severe respiratory infections [5].

Costs attributable to viral respiratory tract infections in both outpatient and inpatient settings are an important burden on national healthcare budgets [5]. Children from poor socio-economic backgrounds are more susceptible to viral respiratory tract infection, as are malnourished children [6]. Overcrowding, especially among children attending day care centres, lack of breastfeeding, poor weaning methods, and exposure of children to passive smoking by their parents are other factors associated with viral respiratory infection [6]. Other important factors are the immunization status of the children as well as the human immunodeficiency virus (HIV) infection status [6, 7].

Respiratory viruses are generally transmitted through inhalation of aerosols or direct contact with respiratory secretions. Transmission is often associated with climatic factors such as low temperatures, low ultraviolet radiation and low humidity which prolong the survival of respiratory viruses in the environment [8]. The seasonality of respiratory viral infections in temperate countries is associated with temperature changes [8]. This can be partly explained by behavioural changes whereby individuals seek shelter and tend to congregate together due to reduced environmental temperature associated with seasonal changes [2]. Viral respiratory infection has also been linked to an increase in susceptibility to bacterial infections by altering physical and immune system barriers leading to increased bacterial super infection [6, 9].

In tropical and subtropical countries, correlation of respiratory viral infections with climatic factors is not well defined, a situation exacerbated by lack of adequate diagnostic facilities [2, 10, 11]. The province of KwaZulu-Natal, in the eastern region of South Africa is defined as having a sub-tropical climate [12] and it is also the epicentre of the HIV epidemic in the country [13]. The aim of this study was to determine the most common viral pathogens associated with ARTI among children between 0 and 5 years of age in KwaZulu-Natal, to describe seasonal patterns for identified viral pathogens, to assess the effect of HIV status on viral respiratory disease pattern, and the impact HIV status has on respiratory cytomegalovirus (CMV) infection. We also investigated the association of CMV and HIV co-infection on viral respiratory infection. A detailed understanding of the prevalence, seasonality and interactions between viral respiratory pathogens would form the basis for the development of public health interventions to prevent associated morbidity and mortality.

## Methods

### Study design

This study involved retrospective data mining of a laboratory information database system. The study population consisted of patients between 0 and 5 years of age whose lower or upper respiratory tract specimens were sent to the National Health Laboratory Services (NHLS) at Inkosi Albert Luthuli Central Hospital (IALCH) in Durban, KwaZulu-Natal, South Africa.

### Specimen types and test methods

Upper respiratory tract samples were either nasopharyngeal swabs or aspirates while lower tract specimens were bronchoalveolar lavages, tracheal aspirates, or endotracheal aspirates. Respiratory specimens were used for both respiratory multiplex and CMV respiratory tests. The samples were collected between 1st January 2011 and 31st July 2015. Laboratory analysis for the respiratory specimens was performed using the multiplex Fast Track Diagnosis (FTD) respiratory pathogens 21 polymerase chain reaction (PCR) test kit (Fast Track Diagnostics, Luxembourg City, Luxembourg). At the IALCH virology laboratory, this kit has been validated for the detection of adenovirus (ADV), enterovirus (EV), influenza A (FluA), influenza B (FluB), human bocavirus (HBoV), human metapneumovirus (HMPV), parainfluenza viruses 1–4 (PIV 1–4), human rhinovirus (Hrhino) and respiratory syncytial virus (RSV) only and therefore these were the pathogens evaluated in this study.

CMV was tested for using the CMV R-gene PCR kit (Biomerieux SA Marcy-l’Étoile, France) while blood specimens were used for HIV testing either by Abbott Architect i4000 ELISA (Abbott, IL, USA) or *Cobas AmpliPrep/Cobas TaqMan* HIV-1 Test (*CAP/CTM*) (Roche Diagnostics) for screening. In children less than 18 months HIV confirmatory testing was conducted using *Cobas AmpliPrep/Cobas TaqMan* HIV-1 Test (*CAP/CTM*) (Roche Diagnostics) and for children older than 18 months of age, Roche Cobas 6000 (Roche diagnostics) was used if the previous HIV test result was positive. Non-viral pathogens (e.g bacteria and fungi) were detected using appropriate culture media.

In this study, NHLS data was collected retrospectively by retrieving test results from the corporate data warehouse (CDW). Information retrieved included demographic and clinical data such as age, sex, specimen type, date of specimen collection, unique hospital number, location of patient in the health facility, respiratory multiplex, HIV, CMV and non-viral isolate test results.

### Statistical analysis

The data retrieved was cleaned by discarding duplicated viral pathogen test results for the same patient within a two-week period only using the first positive results and removing the second duplicated positive results. Laboratory results with the following missing data were excluded: date of birth, specimen type, date of specimen collection and test set requested. Continuous variables such as age were summarised using mean ± standard deviation or median (IQR) and categorical variables such as sex, age groups, facility types, respiratory multiplex and CMV results were summarized using proportions and percentages. We carried out sub-group analysis to determine the between groups *p* value and on the basis of the between groups p value, we conducted pair wise comparisons for all the sub-group pairs while adjusting the alpha level using a Bonferroni correction. The effect of HIV and CMV on viral respiratory infection was investigated by comparing the proportion of respiratory specimens with HIV and CMV coinfection compared with specimens that were HIV and CMV negative using a z test. Categorical variables were compared using Pearson’s chi-squared test or Fisher’s exact test, as appropriate. All analysis was conducted using IBM SPSS version 25 (IBM Corp. Released 2018. IBM SPSS Statistics for Windows, Version 25.0. Armonk, NY: IBM Corp). The level of significance was set at *p* < 0.05.

An objective of the study was to identify and describe seasonal patterns of respiratory viruses using the Autoregressive Integrated Moving Averages (ARIMA) model. ARIMA models are generalisations of Autoregressive Moving Averages and these models are fitted to time series data to understand the data and predict future points in the series [14]. In this study, ARIMA models were used to isolate the seasonal component by removing the underlying trend [15]. The trend was estimated by means of a centred 12 point moving averages. The resulting values were averaged for each month over the duration of the study and expressed as percentages. The 12 percentages were taken as representing the seasonal profile of each respiratory virus. Autocorrelation Function (ACF) and Partial Correlation Function (PACF) plots were used to identify the number of autoregressive and moving average terms, thereby assisting in determining the stationarity and seasonality of the time series. Seasonal indices were calculated as a measure of how the prevalence of the respiratory viruses changed during a given season compared with the season’s average. A seasonal index is a measure of how the prevalence of a respiratory virus compares with the season’s average.

### Ethical considerations

The protocol for the study was approved by the University of KwaZulu-Natal Biomedical Research Ethics Committee (BREC-BCA 143/09), while approval was obtained from the National Health Laboratory Services (NHLS) for the use of the data.

## Results

### Demographic distribution and specimen characteristics

Out of 2172 respiratory specimens during the period under review, 932 (42.9%) came from females and 1175 (54.1%) from males and the remaining 65 (3.0%) specimens did not indicate gender from which they came. The age range of patients studied, were from 0 to 60 months. The median age was 3.0 months, with an interquartile range (IQR) of 1–7 months, with the majority of patients 1599 (73.6%) aged 0 to 6 months.

One thousand nine hundred and forty-nine (89.7%) specimens were from the lower respiratory tract, with 223 (10.3%) upper respiratory specimens. One thousand eight hundred and twenty-three (83.9%) had results available for the multiplex viral respiratory pathogens PCR, with 834 (45.7%) positive and 989 (54.3%) negative (Table 1). The majority of the specimens, 1678 (77.3%) were from patients admitted to the intensive care unit (ICU), 454 (20.9%) specimens were from general hospital ward patients, 38 (1.7%) were from nursery and 2 (0.1%) were from the out-patient department (OPD) *(*Table 1).

**Table 1:** Demographic distribution and specimen characteristics

Variables	*N*	%
Male	1175	54.1
Female	932	42.9
Gender not stated	65	3.0
Total	2172	100
Age (months)		
0–6	1599	73.6
7–12	232	10.7
13–24	204	9.4
25–60	137	6.3
Total	2172	100
Facility type^a^		
District	245	11.3
Tertiary	374	17.2
Specialised	1553	71.5
Total	2172	100
Respiratory multiplex results		
Positive	834	45.7
Negative	989	54.3
Total	1823	100
CMV results		
Positive	416	58.3
Negative	298	41.7
Total	714	100

^a^In South Africa health facilities are categorised into district, tertiary and specialised according to the level of care

**Fig. 1:**
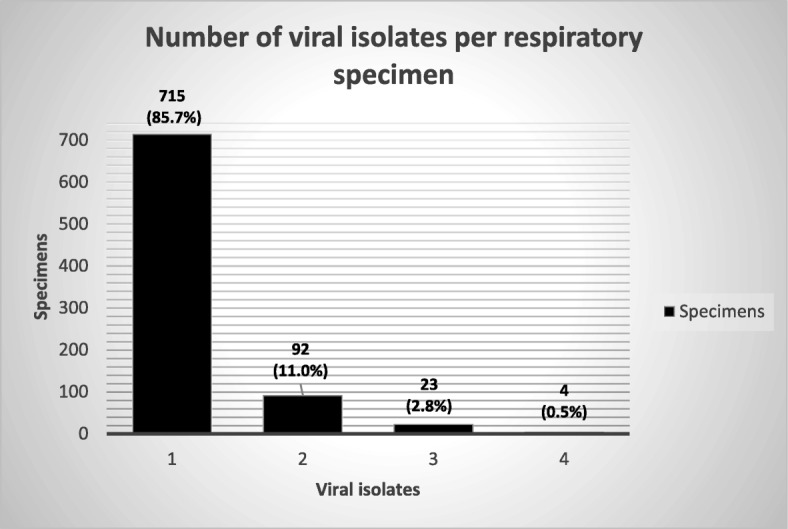
Number of viral isolates

**Fig. 2:**
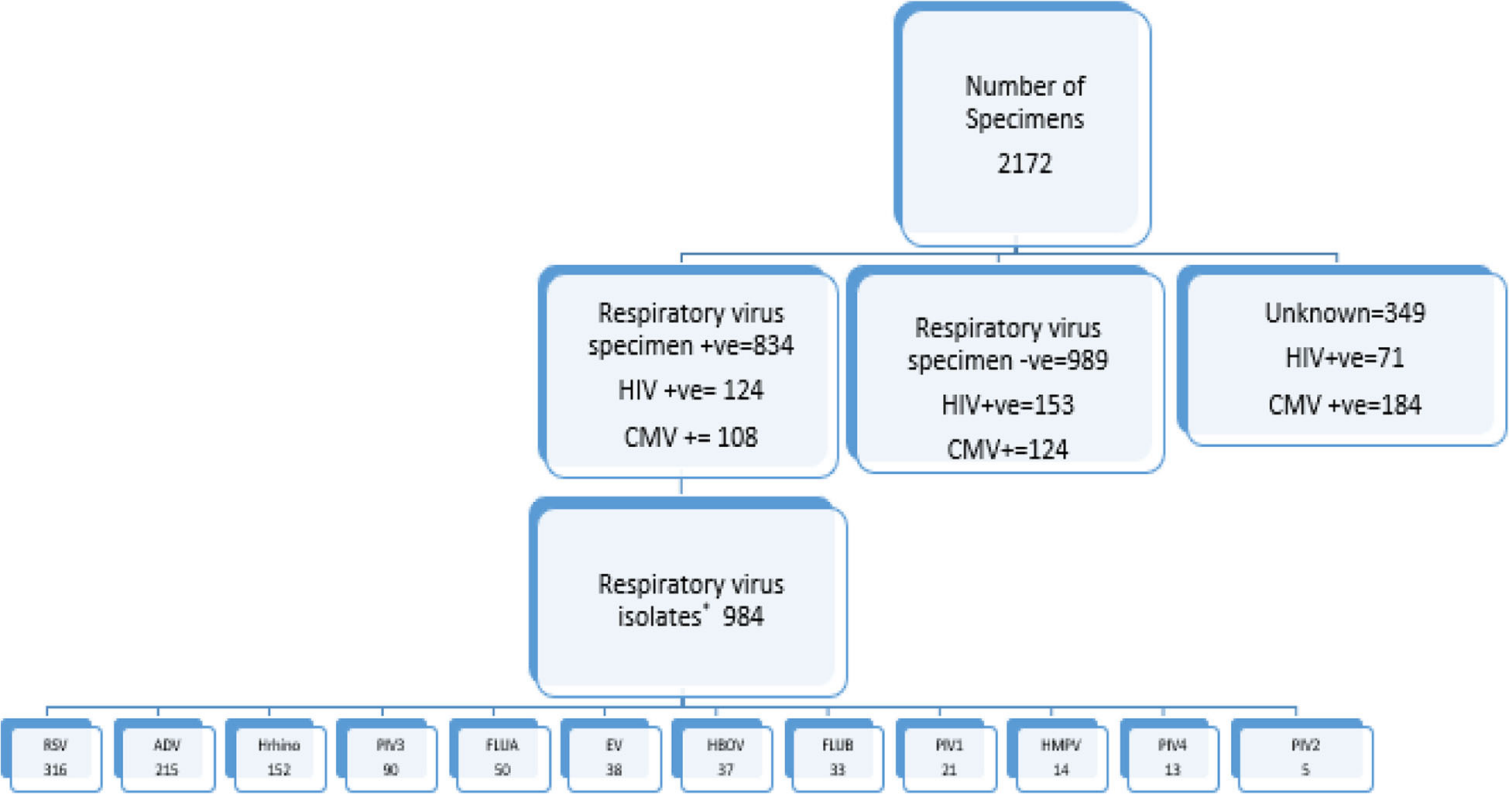
Flow chart of specimen results from those aged ≤5 years old used in the study. *some respiratory virus had more than one isolate

A total of 984 viral pathogens were isolated from 834 positive specimens analysed for respiratory pathogens, out of which 715 (85.7%) had only one viral isolate, 92 (11.0%) had two isolates, 23 (2.8%) had three isolates and 4 (0.5%) possessed four different isolated viruses (Fig. 1). RSV was the most frequently detected virus pathogen in 316 (32.1%) isolates, followed by ADV in 215 (21.8%), Hrhino viruses in 152 (15.4%), PIV3 virus in 90 (9.1%), FluA in 50 (5.1%), FluB in 33 (3.4%) and PIV2 was the least common of the viruses detected, found in only 5 (0.5%) of isolates *(*Fig. 2*).*

Out of the total 2172 specimens, 814 (37.5%) had non-viral isolates, in which *Klebsiella pneumoniae was* the most common isolated non-viral isolate detected in 190 (23.3%), followed by *Staphylococcus aureus* in 108 (13.3%), *Acinetobacter baumannii* in 104 (12.8%), *Candida albicans* in 45 (5.5%), *Pseudomonas aeruginosa* in 56 (6.9%) and *Streptococcus pneumoniae* in 29 (3.6%).

Out of 984 viral pathogens, 579 (58.8%) were from HIV negative individuals, 142 (14.4%) were from HIV positive individuals, while the rest 263 (26.7%) were of unknown HIV status. Five hundred and ninety-nine (60.9%) out of the total 984 viral pathogens were from patients between the ages of 0–6 months, 326 (54.4%) were males and 261 (43.6%) were females and the remaining 12 (2.0%) were of unknown gender *(*Table 2*).* HIV results were available for 1475 (67.9%) specimens with 348 (23.6%) positive and 1127 (76.4%) negative, with the remaining 697 (32.1%) of unknown HIV result. There were only 714 specimens with CMV data available of which 416 (58.3%) were positive. Out of 1475 specimens with HIV results 536 (36.3%) had both CMV and HIV results available, of these 161 (84.7%) were both CMV positive and HIV positive. One hundred and sixty eight (48.6%) were CMV positive and HIV negative, 178 (51.4%) were both CMV negative and HIV negative and 29 (15.3%) were CMV negative and HIV positive. Using a chi-square test a statistically significant association was found between CMV and HIV infection (*p* = 0.0001). This indicates that HIV positive results are more likely to be associated with CMV positive results*.*

**Table 2:** Classification of respiratory viruses according to age, sex, HIV and CMV results

Virus	RSV *n* (%)	ADV *n* (%)	Hrhino *n* (%)	PIV3 *n* (%)	FluA *n* (%)	EV *n* (%)	HBoV *n* (%)	FluB *n* (%)	PIV1 *n* (%)	HMPV *n* (%)	PIV4 *n* (%)	PIV2 *n* (%)	Total	*P* Value
Age														
0–6	250 (79.1)	102 (47.4)	91 (59.9)	59 (65.6)	27 (54)	22 (57.9)	9 (24.3)	15 (45.5)	12 (57.1)	3 (21.4)	6 (46.2)	3 (60)	599 (60.9)	< 0.0001
7–12	10 (3.2)	25 (11.6)	11 (7.2)	9 (10)	3 (6)	4 (10.5)	8 (21.6)	2 (6.1)	4 (19)	3 (21.4)	2 (15.4)	0 (0)	81 (8.2)	
13–24	49 (15.5)	72 (33.5)	40 (26.3)	16 (17.8)	17 (34.0)	6 (23.7)	18 (48.6)	14 (42.4)	4 (19.0)	7 (50.0)	5 (38.5)	2 (40.0)	253 (25.7)	
25–60	7 (2.2)	16 (7.4)	10 (6.6)	6 (6.7)	3 (6)	3 (7.9)	2 (5.4)	2 (6.1)	1 (4.8)	1 (7.1)	0 (0)	0 (0)	51 (5.2)	
Total	316	215	152	90	50	38	37	33	21	14	13	5	984	
Sex														
Male	181 (58.6)	128 (60.7)	75 (50.7)	39 (43.8)	27 (55.1)	26 (70.3)	20 (54.1)	19 (57.6)	10 (50.0)	5 (35.7)	5 (41.7)	2 (40)	537 (55.7)	0.08
Female	128 (41.4)	83 (39.3)	73 (49.3)	50 (56.2)	22 (44.9)	11 (29.7)	17 (45.9)	14 (42.4)	10 (50.0)	9 (64.3)	7 (58.3)	3 (60)	427 (44.3)	
Total	309	211	148	89	49	37	37	33	20	14	12	5	964	
Facility type														
District	30 (9.5)	24 (11.1)	19 (12.5)	14 (15.6)	7 (14.0)	2 (5.3)	4 (10.8)	5 (15.2)	0 (0.0)	1 (7.1)	0 (0.0)	2 (40.0)	108 (11.0)	0.84
Tertiary	54 (17.1)	33 (15.3)	25 (16.4)	17 (18.9)	10 (20.0)	8 (21.1)	3 (8.1)	3 (9.1)	8 (38.1)	3 (21.4)	8 (61.5)	1 (20.0)	173 (17.6)	
Specialised	232 (73.4)	158 (73.5)	108 (71.1)	59 (65.6)	33 (66.0)	28 (73.7)	30 (81.1)	25 (75.8)	13 (61.9)	10 (71.4)	5 (38.5)	2 (40.0)	703 (71.4)	
Total	316	215	152	90	50	38	37	33	21	14	13	5	984	
HIV Positive	27 (11.6)	33 (21.0)	29 (25.2)	20 (29.0)	4 (12.1)	4 (17.4)	7 (24.1)	8 (30.8)	6 (40.0)	2 (16.7)	2 (25.0)	0 (0.0)	142 (19.7)	0.006
HIV negative	205 (88.4)	124 (79.0)	86 (74.8)	49 (71.0)	29 (87.9)	19 (82.6)	22 (75.9)	18 (69.2)	9 (60.0)	10 (83.3)	6 (75.0)	2 (100.0)	579 (80.3)	
Total	232	157	115	69	33	23	29	26	15	12	8	2	721	
CMV Positive	28 (41.8)	29 (74.4)	21 (58.3)	14 (70.0)	9 (50.0)	5 (71.4)	9 (81.8)	5 (62.5)	4 (80.0)	2 (66.7)	7 (100.0)	1 (100.0)	134 (60.4)	0.042
CMV negative	39 (58.2)	10 (25.6)	15 (41.7)	6 (30.0)	9 (50.0)	2 (28.6)	2 (18.2)	3 (37.5)	1 (20.0)	1 (33.3)	0 (0.0)	0 (0.0)	88 (39.6)	
Total	67	39	36	20	18	7	11	8	5	3	7	1	222	

An investigation into the relationship between the presence of respiratory viruses, age, sex, HIV and CMV results using a one-way analysis-of-variance (ANOVA), revealed that there was a statistically significant difference between the four age groups (0–6, 7–12, 13–24 and 25–60 months) with respect to the frequency of respiratory viruses (*p* < 0.0001). There was a statistically higher proportion of ADV results that were coinfected with CMV and HIV than specimens that were not coinfected with CMV and HIV, 5.1 and 0.5% respectively (*p* = 0.004) suggesting an association between ADV and coinfection with CMV and HIV. However, a different picture was observed for RSV, where CMV and HIV negative associated results had higher proportion of RSV compared to coinfected CMV and HIV results (10.4 and 1.9% respectively, *p* = 0.001). In the case of FluA and Hrhino there was no statistically significant difference in the proportion found between CMV and HIV coinfection with *p* values of 0.91 and 0.93 respectively.

The youngest group aged between 0 and 6 months demonstrated the highest number of viral isolates detected at 599 (60.9%), out of the total number of 984 specimens with at least one isolate detected. There was no statistically significant difference in frequency of respiratory viruses between males and females comparing all the age groups (*p* = 0.08).

### Seasonality

**Fig. 3:**
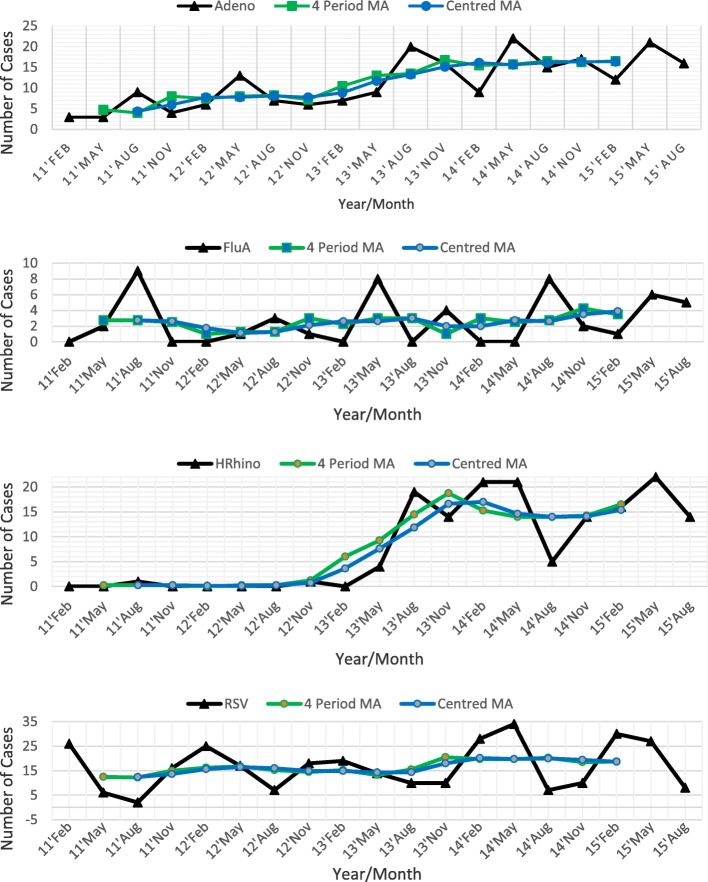
Pattern of viral respiratory tract infections in KwaZulu-Natal: Quaterly distribution and time trends

South Africa has 4 annual seasons, namely autumn, winter, spring and summer [16]. Figure 3 shows the pattern of viral respiratory pathogen isolated during the study period between 1st January 2011 and 31st July 2015. A seasonal time series pattern was observed for ADV (winter peak in August), EV (autumn peak in May), HBoV (summer peak in February), PIV1 (spring peak in November) and PIV3 (spring peak in November). Stationary or untrended seasonal variation was observed for FluA (winter peak in August) and RSV (summer peak in February). Irregular cyclical time series trends were observed for HMPV, PIV2 and Hrhino, where the trends exhibited rises and falls that were not of fixed period. A seasonal time series pattern is characterised by a regular and predictable change that occurs every calendar year, while stationary or untrended seasonal variation is characterised by a constant seasonal variation that neither increases or decreases over time.

Seasonal indices are shown in Table 3. A seasonal index is a measure of how the prevalence of a respiratory virus compares with the season’s average. It shows that in autumn and winter, ADV was detected 1.287 and 1.340 times more than the average. An autumn seasonal index of 2.353 for PIV4, indicates that in autumn more than twice the average prevalence of PIV4 was observed. Based on seasonal indices, all the viruses demonstrated a seasonal spread, with some viruses detected two seasons per year (a biannual pattern), such as ADV (autumn and winter), FluA (autumn and winter), HMPV (summer and spring), PIV3 (summer and spring) and RSV (summer and autumn).

**Table 3:** Seasonal indices

Season	Seasonal Indices											
	ADV	EV	FluA	FluB	HBOV	HMPV	PIV1	PIV2	PIV3	PIV4	Hrhino	RSV
Summer	0.713	**1.011** ^a^	0	0	**2.471** ^a^	**1.714** ^a^	0.686	0.667	**1.112** ^a^	0.571	0.569	**1.450** ^a^
Autumn	***1.287*** ^a^	***1.449*** ^a^	**1.312** ^a^	0	**1.000**	0	**1.067** ^a^	0	0.480	**2.353** ^a^	0.653	**1.450** ^a^
Winter	***1.340*** ^a^	0.458	**2.180** ^a^	0.841	0	0.533	0.741	0	0.875	0.308	**1.489** ^a^	0.412
Spring	0.885	0.695	0.761	**1.844** ^a^	0.242	**1.949** ^a^	**2.115** ^a^	0.889	**1.650** ^a^	0.308	0.792	0.864

^a^Boldface indicates Seasonal indices above 1 means that prevalence of a respiratory virus is above average

## Discussion

Viral agents play an important role in respiratory infections associated with disease in young children but their prevalence, seasonality and predisposing factors are not well understood in resource-poor countries. The results in this study show that RSV was the most commonly detected viral pathogen in the respiratory specimens, consistent with the view that RSV is a leading cause of respiratory tract infection in infants and young children worldwide [10] causing an estimated 66,000 to 199,000 deaths per year globally in children less than 5 years of age [17]. The overall prevalence of RSV (32.1%) is comparable to previous studies done in other developing countries with tropical and sub-tropical climates such as Ghana [10] and Malaysia [8] though in a South African study conducted in Pretoria [18], RSV was more common in HIV-uninfected children than in HIV-infected children which was consistent with our study.

ADV was the second most commonly detected virus (21.8%) in this study, similar to a Ghanaian study although the prevalence was lower at 10.2% [10]. A Malaysian study also found ADV to be one of the most common respiratory viral isolates, although it ranked fourth in that study [8]. In another South African study done in Cape Town [19], ADV respiratory infections was isolated in 10.9% of all respiratory tract samples tested and it was linked to severe morbidity with 36.9% needing ICU admission and 14.1% developing persistent lung disease. The latter study is comparable to our study where 66.0% of the specimens were from the ICU which is an indirect indicator of disease severity.

Hrhino virus was the third most commonly isolated pathogen in this study, in contrast to studies by Pretorius et al. (2012) and Annamalay et al. (2016) in which it was commonest [11, 18]. Both studies highlight that Hrhino virus is an important viral pathogen in children in the South African setting. In the Annamalay et al. (2016) study Hrhino virus detection was highest in the 18–24 months age group [18] compared to our study where it was commonest in the age group 0–6 months. In a study by Abadom et al. (2016) in South Africa, HIV was more prevalent among cases of influenza associated with severe acute respiratory infection [20]. However, this is different in our study, where most of the specimens with a positive influenza result were linked to an HIV negative result 29 (87.9%) compared to an HIV positive result 4 (12.1%).

Out of all the detected viral pathogens 599 (60.9%) were isolated from the age group 0–6 months, emphasizing the high infection burden in this group and likely associated morbidity and mortality, similar to a study by Khor et al. (2012, Malaysia) where 76.2% of the positive cases were isolated from children less than 1 year old [8]. Cytomegalovirus has been implicated as a cause of increased morbidity and mortality and associated with respiratory disease, especially in immunocompromised individuals such as those infected with HIV, transplant patients and patients on therapy for autoimmune diseases [21–23]. In this current study, there was significant association between coinfected HIV and CMV results which is similar to a study conducted by Zampoli et al. (2011) in Cape Town where CMV associated respiratory disease was more common in HIV infected than uninfected children [21].

An important finding from our study is that most viral pathogens detected displayed seasonal prevalence trends, with most having peak periods between autumn and winter, suggestive of increased susceptibility to respiratory viral infections during the colder months. Overall, these results are consistent with other studies from Malaysia, Brazil and South Africa that all indicate that seasonality is a common feature of viral respiratory infections [2, 8, 11]. However, there are some contrasting findings between our study and other studies, such as a study conducted in Malaysia were no seasonal trend was observed for ADV [8]. Some studies have also documented that ADV is normally isolated all year round with no distinct seasonal trends [24].

Hrhino virus was isolated all year round with the trends exhibiting rises and falls that were not of fixed period in our study which is different from a study by Gardinassi et al. (2012), that was conducted in Brazil where outbreaks were observed in spring, autumn and winter [2]. In our study a seasonality pattern was noted for FluA from 2011 to 2015, with first yearly isolations in autumn and a peak in winter, which is similar to previous surveillance reports where the virus was first isolated in autumn, peaked in winter and tapered off in late winter [25–29]. However, in 2015, more Flu B than Flu A was detected in our study, which is similar to the influenza-like illness (ILI) surveillance report by NICD [29] and this could be an emerging trend in the prevalence of Flu B.

The limitations of our study could be due to the fact that it was retrospective in nature and therefore it was not possible to differentiate between community acquired and nosocomial infections. Emerging respiratory viruses were not tested for in this study, which can also pose a significant public health risk especially in children with immature immune systems. In the same vein, inferring whether a pathogen was a bystander or contributing to disease was a challenge in our study due to the probability of patients having other co-morbidities and therefore more detailed epidemiological and clinical studies are required to evaluate the relative importance of respiratory viral pathogens in this setting. The diagnostic kit used for detection of viral infections was also not exhaustive, and therefore important viral infections that may contribute to morbidity and mortality in children may have been missed.

## Conclusions

Viruses play an important role in respiratory diseases in young children and this report shows the high burden of infection in children especially the younger age group of 0 to 6 months. The association between HIV infected children and CMV respiratory infection highlights the importance of investigating CMV in sick young children.

The data on seasonality shows that most viral respiratory pathogens showed seasonal patterns with slight differences from other studies with pathogens such as ADV previously thought to show no seasonal pattern showing regular predictable peaks and trends in this study. Our study highlights the need for more comprehensive studies on viral associated respiratory tract infections with the goal of developing more effective interventional strategies to prevent and treat these infections that impose a huge public health and socioeconomic burden in resource-limited countries. Overall, more comprehensive studies are needed to identify prevalence and seasonal trends of respiratory viral agents relevant to developing countries.

## Abbreviations

ADV: Adenovirus
ARTI: Acute respiratory tract infection
CDW: Corporate data warehouse
CMV: Cytomegalovirus
EV: Enterovirus
FluA: Influenza A
FluB: Influenza B
HBoV: Human boca virus
HIV: Human immunodeficiency virus
HMPV: Human metapneumovirus
Hrhino: Human Rhino virus
ICU: Intensive care unit
IFA: Immunofluorescence assay
NHLS: National Health Laboratory Services
PCR: Polymerase chain reaction
PIV1: Parainfluenza virus 1
PIV2: Parainfluenza virus 2
PIV3: Parainfluenza virus 3
PIV4: Parainfluenza virus 4
RSV: Respiratory syncytial virus

## References

[1] McLean HQ, Peterson SH, King JP, Meece JK, Belongia EA. School absenteeism among school-aged children with medically attended acute viral respiratory illness during three influenza seasons, 2012-2013 through 2014-2015. Influenza Other Respir Viruses. 2017;11(3):220–9. https://www.ncbi.nlm.nih.gov/pmc/articles/PMC5410714/pdf/IRV-11-220.pdf. Accessed 21 May 2018 10.1111/irv.12440 PMC5410714 27885805

[2] Gardinassi LG, Simas PV, Salomão JB, Durigon EL, Trevisan DM, Cordeiro JA, Lacerda MN, Rahal P, Souza FP. Seasonality of viral respiratory infections in southeast of Brazil: the influence of temperature and air humidity. Braz J Microbiol. 2012;43(1):98–108. 10.1590/S1517-838220120001000011 PMC3768995 24031808

[3] Wong-Chew RM, Espinoza MA, Taboada B, Aponte FE, Arias-Ortiz MA, Monge-Martínez J, Rodríguez-Vázquez R, Díaz-Hernández F, Zárate-Vidal F, Santos-Preciado JI, López S (2015). Prevalence of respiratory virus in symptomatic children in private physician office settings in five communities of the state of Veracruz, Mexico. BMC research notes.

[4] World Health Organization. World Health Statistics 2018: Monitoring health for the sustainable development goals. http://apps.who.int/iris/bitstream/handle/10665/272596/9789241565585-eng.pdf?ua=1. Accessed 9 June 2018.

[5] Van Woensel JB, Van Aalderen WM, Kimpen JL (2003). Viral lower respiratory tract infection in infants and young children. BMJ: British Medical Journal.

[6] Ujunwa FA, Ezeonu CT (2014). Risk factors for acute respiratory tract infections in under-five children in Enugu Southeast Nigeria. Annals of medical and health sciences research.

[7] Lonngren C, Morrow BM, Haynes S, Yusri T, Vyas H, Argent AC (2014). North–south divide: distribution and outcome of respiratory viral infections in paediatric intensive care units in Cape Town (South Africa) and Nottingham (United Kingdom). J Paediatr Child Health.

[8] Khor CS, Sam IC, Hooi PS, Quek KF, Chan YF (2012). Epidemiology and seasonality of respiratory viral infections in hospitalized children in Kuala Lumpur, Malaysia: a retrospective study of 27 years. BMC pediatrics.

[9] Tregoning JS, Schwarze J (2010). Respiratory viral infections in infants: causes, clinical symptoms, virology, and immunology. Clin Microbiol Rev.

[10] Kwofie TB, Anane YA, Nkrumah B, Annan A, Nguah SB, Owusu M (2012). Respiratory viruses in children hospitalized for acute lower respiratory tract infection in Ghana. Virol J.

[11] Pretorius MA, Madhi SA, Cohen C, Naidoo D, Groome M, Moyes J, Buys A, Walaza S, Dawood H, Chhagan M, Haffjee S (2012). Respiratory viral coinfections identified by a 10-plex real-time reverse-transcription polymerase chain reaction assay in patients hospitalized with severe acute respiratory illness—South Africa, 2009–2010. J Infect Dis.

[12] Medical education partner initiative (MEPI), University of KwaZulu-Natal. *Geography-South* Africa*:* http://mepi.ukzn.ac.za/OtherInfo/Geographyaspx. Accessed 15 July 2017.

[13] KwaZulu-Natal, Department of health (2010). HIV counselling and testing campaign (HCT) in KwaZulu-Natal.

[14] Helfenstein U (1986). Box-Jenkins modelling of some viral infectious diseases. Stat Med.

[15] Chadsuthi S, Iamsirithaworn S, Triampo W, Modchang C. Modeling seasonal influenza transmission and its association with climate factors in Thailand using time-series and ARIMAX analyses. Computational and mathematical methods in medicine. 2015;2015 10.1155/2015/436495 PMC4667155 26664492

[16] Department of Environmental Affairs. South African weather services (SAWS). http://www.weathersa.co.za/learning/weather-questions/82-how-are-the-dates-of-the-four-seasons-worked-out. Accessed 18 July 2017.

[17] Mazur NI, Bont L, Cohen AL, Cohen C, Von Gottberg A, Groome MJ, Hellferscee O, Klipstein-Grobusch K, Mekgoe O, Naby F, Moyes J (2016). Severity of respiratory syncytial virus lower respiratory tract infection with viral coinfection in HIV-uninfected children. Clin Infect Dis.

[18] Annamalay AA, Abbott S, Sikazwe C, Khoo SK, Bizzintino J, Zhang G, Laing I, Chidlow GR, Smith DW, Gern J, Goldblatt J (2016). Respiratory viruses in young south African children with acute lower respiratory infections and interactions with HIV. J Clin Virol.

[19] Zampoli M, Mukuddem-Sablay Z (2017). Adenovirus-associated pneumonia in south African children: presentation, clinical course and outcome. SAMJ: South African Medical Journal.

[20] Abadom TR, Smith AD, Tempia S, Madhi SA, Cohen C, Cohen AL (2016). Risk factors associated with hospitalisation for influenza-associated severe acute respiratory illness in South Africa: a case-population study. Vaccine.

[21] Zampoli M, Morrow B, Hsiao NY, Whitelaw A, Zar HJ (2011). Prevalence and outcome of cytomegalovirus-associated pneumonia in relation to human immunodeficiency virus infection. Pediatr Infect Dis J.

[22] Govender K, Jeena P, Parboosing R (2017). Clinical utility of bronchoalveolar lavage cytomegalovirus viral loads in the diagnosis of cytomegalovirus pneumonitis in infants. J Med Virol.

[23] Adland E, Klenerman P, Goulder P, Matthews P (2015). Ongoing burden of disease and mortality from HIV/CMV coinfection in Africa in the antiretroviral therapy era. Front Microbiol.

[24] Richman DD, Whitley RJ, Hayden FG. Clinical virology 4th edition ed. Washington DC: ASM press; 2017. Pg 9.

[25] National Health Laboratory Services (NHLS), *Communicable diseases surveillance bulletin*. National institute for communicable diseases (NICD). 2011. http://www.nicd.ac.za/assets/files/CommDisBull%2010(2)-May%20final2012.pdf. Accessed 17 Feb 2016.

[26] National Health Laboratory Services (NHLS), *Communicable diseases surveillance bulletin*. National institute for communicable diseases 2012 http://www.nicd.ac.za/assets/files/Communicable%20Diseases%20Surveillance%20Bulletin%20April%202013.pdf. Accessed 17 Feb 2016.

[27] National Health Laboratory Services (NHLS), *Communicable diseases surveillance bulletin*. National institute for communicable diseases 2013 http://www.nicd.ac.za/assets/files/CommDisBull%2012(1)-April%202014_Fin.pdf. Accessed 17 Feb 2016.

[28] National Health Laboratory Services (NHLS), *Communicable diseases surveillance bulletin*. National institute for communicable diseases 2014 http://www.nicd.ac.za/assets/files/CommDisBull%2013(1)-April%202015.pdf. Accessed 17 Feb 2016.

[29] National Health Laboratory Services (NHLS), *Communicable diseases surveillance bulletin*. National institute for communicable diseases 2015 http://www.nicd.ac.za/assets/files/CommDisBull%2014(1)-Mar2016(1).pdf. Accessed 17 Feb 2016.

